# Three-year incidence of Nd:YAG capsulotomy and posterior capsule opacification and its relationship to monofocal acrylic IOL biomaterial: a UK Real World Evidence study

**DOI:** 10.1038/s41433-018-0131-2

**Published:** 2018-06-11

**Authors:** Paul G. Ursell, Mukesh Dhariwal, Katarina Majirska, Frank Ender, Shoshannah Kalson-Ray, Alessandra Venerus, Cristiana Miglio, Christine Bouchet

**Affiliations:** 1 Epsom & St Helier University Hospitals NHS Trust, Epsom, Surrey UK; 2 Novartis Ireland Ltd, Dublin, Ireland; 3 Alcon Eye Care Limited, Camberley, Surrey UK; 4 Alcon Management SA, Cointrin-Geneva, Switzerland; 5 IQVIA, London, UK; 6 Alcon laboratories Inc, Fort Worth, TX USA

## Abstract

**Purpose:**

To evaluate 3-year incidence of Nd:YAG capsulotomy and PCO and compare the effect of different IOL materials.

**Methods:**

Data were retrospectively collected from seven UK ophthalmology clinics using Medisoft electronic medical records. Eyes from patients ≥65 years undergoing cataract surgery with implantation of acrylic monofocal IOLs during 2010–2013 and 3-year follow-up were analysed. Nd:YAG capsulotomy and PCO incidence proportions were reported for 3 IOL cohorts: AcrySof, other hydrophobic and hydrophilic acrylic IOLs. Unadjusted/adjusted odds ratios (OR) of Nd:YAG capsulotomy were calculated through logistic regression for non-AcrySof cohorts versus AcrySof. A sub-group analysis in single-piece IOLs (>90% of sample eyes) was also performed.

**Results:**

The AcrySof cohort included 13,329 eyes, non-AcrySof hydrophobic 19,025 and non-AcrySof hydrophilic 19,808. The 3-year Nd:YAG capsulotomy incidence (95% CI) for AcrySof (2.4%, 2.2–2.7%) was approximately two times lower than non-AcrySof hydrophobic IOLs (4.4%, 4.1–4.7%) and approximately fourfold lower than non-AcrySof hydrophilic IOLs (10.9%, 10.5–11.3%). Trends were similar in PCO incidence (AcrySof: 4.7%; non-AcrySof hydrophobic: 6.3%; non-AcrySof hydrophilic: 14.8%). Also in the analysis restricted to single-piece IOLs, the pattern remained (2.4% vs 5.1% vs. 10.9%, respectively). Adjusted regression analysis showed a approximately two and fivefold increased odds of Nd:YAG for non-AcrySof hydrophobic and hydrophilic acrylic IOLs respectively vs. AcrySof IOLs. Nd:YAG capsulotomy ORs were similar and remained statistically significant in the single-piece IOL sub-group.

**Conclusions:**

Real-world evidence shows that within 3 years following implantation, AcrySof IOLs are significantly superior in reducing Nd:YAG capsulotomy and PCO incidence compared to other hydrophilic and hydrophobic acrylic IOLs.

## Introduction

Posterior capsule opacification (PCO) is the most common complication following cataract surgery [1, 2]. It can occur between few months and many years after implantation of intraocular lenses (IOLs), with incidence figures ranging from  < 5% to as high as 50% [1]. Neodymium-doped yttrium aluminium garnet (Nd:YAG) laser capsulotomy is a widely accepted surgical method to treat PCO but is costly and may present a risk of complications [3]. Therefore, measures are usually taken during cataract surgery to prevent or delay the onset of PCO. Currently, among other strategies, there is great interest from surgeons, patients and payers in the prevention of PCO and subsequent Nd:YAG capsulotomy through improvements in IOL material and design [1, 3]. While there are known risk factors such as round vs sharp edge [4], more research is needed to understand the role of IOL material and design characteristics in the PCO formation.

Prospective controlled studies on the incidence of Nd:YAG capsulotomy and PCO by IOL type have been conducted over the past two decades, reporting more favourable outcomes for hydrophobic acrylic IOLs compared to hydrophilic [5–10]. In addition, a 6 mm IOL optic diameter and a sharp optic edge were shown to reduce the development of PCO in randomised clinical trials (RCT) [1]. Furthermore, favourable outcomes were reported in RCTs in support of the use of bio-adhesive AcrySof^®^ material (Alcon laboratories, Inc., Texas, USA) versus other IOL materials [11–15].

Although RCTs are generally considered the gold standard to evaluate comparative effectiveness, it is generally not feasible to provide estimates in large populations or over extended time horizons [16, 17]. Similarly, prospective studies following patients for years or until an outcome of interest may be limited in terms of time and associated cost. Real World Evidence (RWE), studies using high-quality databases can capture medical record information to longitudinally assess long-term outcomes for large cohorts of patients, thus providing robust evidence based on real-world clinical practice. Regulatory bodies such as US Food and Drug Administration (USFDA) and the health technology assessment bodies such as the UK based National Institute for Health and Care Excellence (NICE) are increasingly using real-world evidence to support their decision-making for medical devices [16, 18].

Although previously assessed in single-centre, short duration, and limited sample size prospective clinical studies, robust and longitudinal evidence on the association between IOL material and Nd:YAG capsulotomy to treat PCO remains sparse.

The aim of this RWE study was to evaluate the long-term incidence of Nd:YAG capsulotomy and PCO following age-related cataract surgery, comparing 3-year outcomes for hydrophobic acrylic AcrySof IOLs versus cohorts of other hydrophilic and hydrophobic acrylic monofocal IOLs.

## Methods

### Data source

Data were collected from 7 UK National Health Service (NHS) cataract clinics using the Medisoft Ophthalmology Electronic Medical Record (EMR) system. Medisoft has covered ophthalmic care episodes and diagnostic information for >1 million patients at over 150 UK ophthalmology clinics [19]. It is a validated and widely accepted research data source with a strong portfolio of publications, including numerous RWE studies conducted by the Royal College of Ophthalmologists [20]. All patient data used in this study was fully anonymised and compliant with the UK NHS rules governing use of patient-level healthcare data (as defined in the Data Protection Act of 1998). This study was approved by each of the participating NHS centre’s Caldicott Guardian.

The sites were selected to provide large numbers of procedures, reliable recording of cataract surgery and post-operative follow-up data including record of Nd:YAG capsulotomy in the Medisoft EMR system. Each clinic was interviewed to ensure that all follow-up appointments for PCO diagnosis/YAG capsulotomy would be recorded only at the centre where cataract surgery was performed.

### Study population

Eye-level data recorded between 1 January 2010 and 31 December 2016 were extracted for all eyes undergoing cataract surgery in the selected clinics, with recorded details of the implanted IOL type. After data extraction, all eyes implanted with monofocal acrylic IOLs were included into the study. In order to avoid confounding the study, cohorts with eyes implanted with non-acrylic IOLs or with IOL models implanted rarely, IOLs that were used in less than 100 cases across all clinics, multifocal or toric IOLs were excluded.

In order to be included into the study, eyes were required to have a first record of phacoemulsification of the lens and in-the-bag implantation of a monofocal acrylic intraocular lens between 1 January 2010 and 31 December 2013, to ensure at least 3 years of follow-up data was available in the Medisoft database. This date of cataract surgery and IOL implantation was defined as the index date. Data from patients who died within the follow-up period and whose record of death was present in Medisoft database were excluded from the analysis. As the data used for the study originated from specialist visits in ophthalmology centres, we assumed that if a patient was not recorded as deceased and was not seen in the clinic after the index date, the patient did not experience the outcomes of interest (Nd:YAG capsulotomy or PCO). The selected clinics confirmed that they were the only units in their geographic area performing Nd:YAG capsulotomies and all recorded the procedure onto Medisoft system, minimising the likelihood of loss to follow-up due to transfer to other centres. However, it may be possible that some patients re-located following cataract surgery and their post-operative medical history was lost to follow-up.

In attempts to exclude secondary types of non-age-related cataracts, eyes from patients aged less than 65 years at the date of cataract surgery were not included in the analyses. In addition, eyes were excluded if they had invalid cataract surgery records (e.g. more than one record for the same eye) or if they had a record of co-surgeries or complications pre, post or during cataract surgery (as defined by clinical input), which may impact the incidence of PCO. These comprised of capsular tension ring, vitrectomy and PC rupture.

### Statistical analyses

All analyses were performed at the eye level using SAS^®^ software version 9.4 [21]. The selected eyes were categorised as 1 of 3 different study cohorts according to the optic material and hydro-properties of the implanted acrylic IOLs: AcrySof IOLs, which are hydrophobic; non-AcrySof hydrophobic acrylic IOLs; and non-AcrySof hydrophilic acrylic IOLs. For each cohort, records of Nd:YAG capsulotomy procedure and PCO were used to evaluate the incidence proportions and 95% confidence intervals (CI) over 3 years from index date. Results at shorter time intervals of 1 and 2 years were also reported.

Nd:YAG capsulotomy was identified within EMRs according to the NHS Office of Population Censuses and Surveys Classification of Interventions and Procedures, version 4 (OPCS-4) [22], using the codes C73.3 (capsulotomy of posterior lens capsule) and Y08.6 (YAG posterior capsulotomy). Presence of PCO was identified within the records of post-cataract surgery complications. For Nd:YAG capsulotomy incidence, descriptive survival curves over the 3 years were plotted using the Kaplan–Meier method, whereby the failure event was a record of Nd:YAG capsulotomy procedure. In addition, the unadjusted and adjusted (using a step-wise approach) odds ratios (ORs) and 95% CI of receiving Nd:YAG capsulotomy within the 3 year follow-up period were calculated through multivariate logistic regression, for non-AcrySof hydrophobic acrylic and non-AcrySof hydrophilic acrylic cohorts versus AcrySof IOLs cohort including both multi-piece and single-piece IOLs. The significance level adopted was 0.05.

As IOLs of different haptic design may not produce the same clinical results [23, 24], the Nd:YAG capsulotomy incidence proportions and ORs were also analysed for the largest sub-group of eyes implanted with single-piece acrylic IOLs only (92% of total, across all 3 IOL groups).

## Results

### Population attrition and IOL types included in the study

**Fig. 1:**
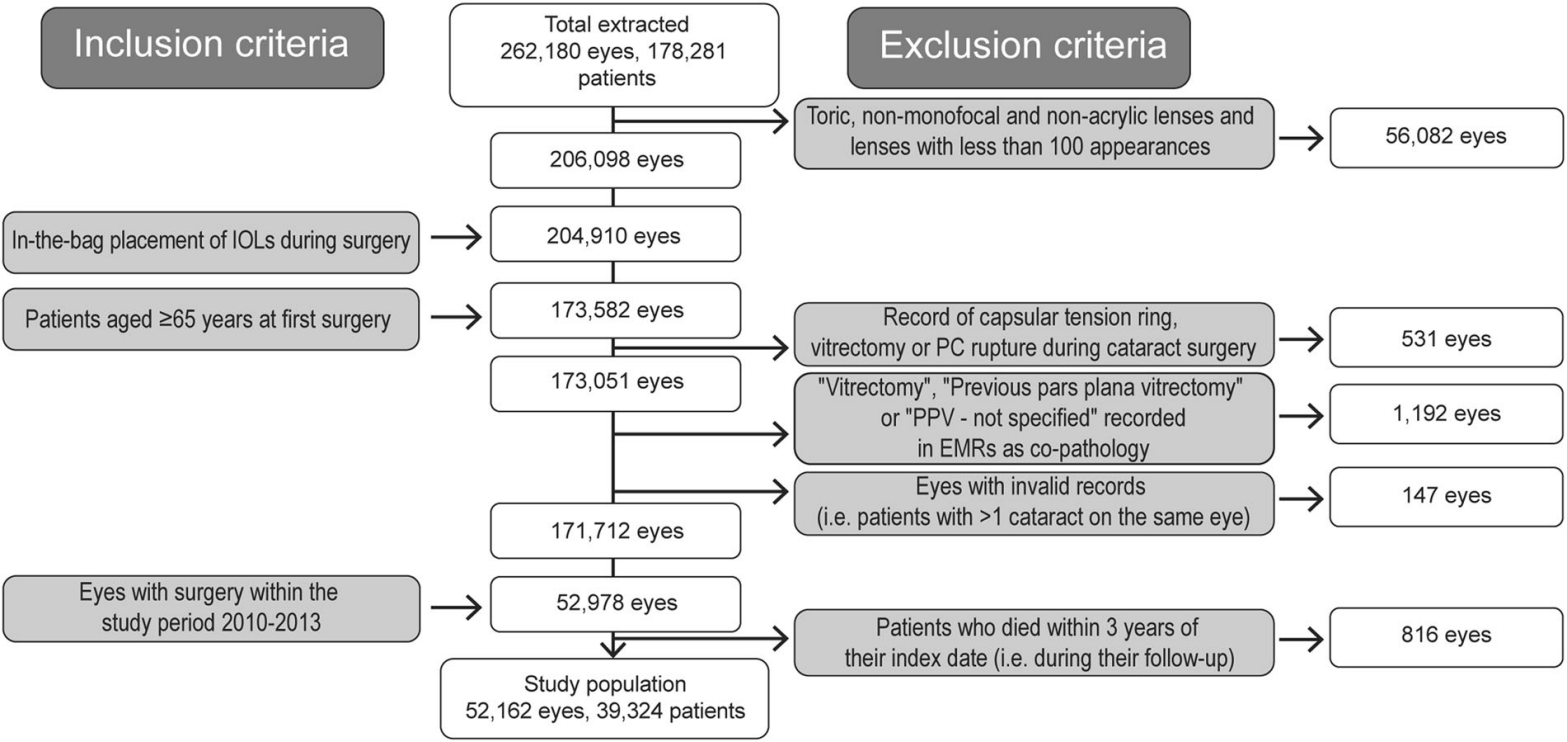
Population selection and attrition for eyes included in the study

Population attrition is shown in Fig. 1. The AcrySof cohort included 13,329 eyes and the non-AcrySof acrylic cohort 38,833 eyes, of which 19,025 were hydrophobic and 19,808 were hydrophilic acrylic IOLs. The IOL types included in each study cohort are described in Table 1, along with their edge (360/non-360 sharp edge) and haptic (single/multi-piece) designs. Overall, 92% of the eyes were implanted with single-piece IOLs and were included in the sub-analysis. In the AcrySof cohort, 98% were single-piece IOLs with a non-360° sharp edge. In the hydrophilic cohort 100% were single-piece with a 360° sharp edge. In the non-AcrySof hydrophobic cohort, 79% were single-piece with a 360° sharp edge.

**Table 1:** Number of eyes included in each of the study groups by IOL model and design

Optic material^a^	IOL model	Haptic design	Surface design	*N*
AcrySof Hydrophobic (*N* = 13,329)	SA60AT (Alcon)	Single-piece	Non-360 sharp edge	6793
	SN60WF IQ (Alcon)	Single-piece	Non-360 sharp edge	6077
	NATURAL (Alcon)	Single-piece	Non-360 sharp edge	141
	MA60AC (Alcon)	Multi-piece	Non-360 sharp edge	166
	MA30AC (Alcon)	Multi-piece	Non-360 sharp edge	5
	MA50BM (Alcon)	Multi-piece	360 sharp edge	99
	MA60MA (Alcon)	Multi-piece	360 sharp edge	47
	MA60BM (Alcon)	Multi-piece	360 sharp edge	1
Non-AcrySof Hydrophobic (*N* = 19,025)	TECNIS ZCB00 (AMO)	Single-piece	360 sharp edge	15,083
	SENSAR AR40E (AMO)	Multi-piece	360 sharp edge	110
	TECNIS ZA9003 (AMO)	Multi-piece	Non-360 sharp edge	3832
Non-AcrySof Hydrophilic (*N* = 19,808)	AKREOS ADAPT (B&L)	Single-piece	360 sharp edge	8514
	SOFTEC HD (Lenstec)	Single-piece	360 sharp edge	4701
	C-FLEX 970 C (Rayner)	Single-piece	360 sharp edge	3607
	SOFTEC 1 (Lenstec)	Single-piece	360 sharp edge	1573
	AKREOS MICS MI60 (B&L)	Single-piece	360 sharp edge	830
	SUPERFLEX 920 H (Rayner)	Single-piece	360 sharp edge	576
	INCISE (Renishaw)	Single-piece	360 sharp edge	6
	570 H (Rayner)	Multi-piece	Non-360 sharp edge	1

^a^Acrylic IOLs are also available as hybrid surface properties (i.e. hydrophilic with hydrophobic surface properties); however, none were identified in the study sample

### Baseline characteristics of the selected population

Patient baseline characteristics are presented in Table 2. Only small differences were observed between the three IOL cohorts, which were accounted for in the logistic regression analysis. Without considering posterior capsular rupture (excluded from the analysis for its potential to impact PCO incidence) and PCO (evaluated separately as study outcome), a small proportion of the included eyes recorded other intraoperative (1.9%, *N* = 969) or post-operative (5.2%, *N* = 2720) complications.

**Table 2:** Baseline characteristics for all eyes and by study groups

Characteristic	Statistic	All eyes *N* = 52,162	AcrySof *N* = 13,329	Non-AcrySof hydrophilic *N* = 19,808	Non-AcrySof hydrophobic *N* = 19,025
*N* of eyes operated					
Single eye operated	*N* (%)	26,484 (50.8)	7466 (56.0)	11,080 (55.9)	7940 (41.7)
Eyes with fellow eye operated	*N* (%)	25,678 (49.2)	5863 (44.0)	8728 (44.1)	11,085 (58.3)
Age at index date for first eyes operated	*N*	39,324 (75.4)	10,606 (79.6)	15,391 (77.7)	13,327 (70.0)
	Mean (SD)	78.7 (7.0)	78.7 (7.1)	78.9 (7.1)	78.5 (7.0)
Age at index date for second eyes operated	*N*	12,838 (24.6)	2723 (20.4)	4417 (22.3)	5698 (30.0)
	Mean (SD)	78.9 (6.7)	78.8 (6.8)	79.0 (6.8)	78.8 (6.7)
Gender	*N* (%) males	20,852 (40.0)	5292 (39.7)	7989 (40.3)	7571 (39.8)
Co-pathologies					
Record of any co-pathology (yes)	*N* (%)	20,343 (39.0)	4743 (35.6)	7792 (39.3)	7808 (41.0)
Disease not specified	*N* (%)	1932 (3.7)	420 (3.2)	844 (4.3)	668 (3.5)
Glaucoma	*N* (% of recorded)	5195 (25.5)	1031 (26.7)	2238 (28.7)	1926 (24.7)
Age-related macular degeneration	*N* (% of recorded)	5101 (25.1)	1213 (25.6)	1693 (21.7)	2195 (28.1)
Diabetic retinopathy	*N* (% of recorded)	2688 (13.2)	783 (16.5)	995 (12.8)	910 (11.7)
Brunescent/white cataract	*N* (% of recorded)	1993 (9.8)	438 (9.2)	988 (12.7)	567 (7.3)
Corneal pathology	*N* (% of recorded)	1787 (8.8)	380 (8.0)	547 (7)	860 (11)
Most recent BDVA prior to surgery (logMAR)	Missing	2203 (4.2)	638 (5.0)	613 (3.1)	952 (5.0)
	Median (IQR)	0.3 (0.2–0.5)	0.3 (0.2–0.5)	0.3 (0.2–0.5)	0.3 (0.2–0.5)
Use of vision blue (yes)	*N* (%)	1830 (3.5)	401 (3.0)	764 (3.9)	665 (3.5)
Pupil size					
Small	*N* (%)	1794 (3.4)	489 (3.7)	696 (3.5)	609 (3.2)
Medium	*N* (%)	7482 (14.3)	2812 (21.1)	2327 (11.7)	2343 (12.3)
Large	*N* (%)	42,886 (82.2)	10,028 (75.2)	16,785 (84.7)	16,073 (84.5)
Incision meridian					
Superior	*N* (%)	21,371 (41.0)	6196 (46.5)	10,544 (53.2)	4631 (24.3)
Temporal	*N* (%)	8663 (16.6)	2384 (17.9)	1532 (7.7)	4747 (25)
On-axis	*N* (%)	22,128 (42.4)	4749 (35.6)	7732 (39)	9647 (50.7)
Incision site					
Scleral	*N* (%)	1713 (3.3)	1240 (9.3)	0 (0)	473 (2.5)
Limbal	*N* (%)	1447 (2.8)	458 (3.4)	297 (1.5)	692 (3.6)
Clear corneal	*N* (%)	49,002 (93.9)	11,631 (87.3)	19,511 (98.5)	17,860 (93.9)
IOL power	Mean (SD)	21.6 (3.8)	21.5 (3.8)	21.2 (3.6)	22.2 (3.8)
Surgeon seniority					
Missing	*N* (%)	99 (0.2)	37 (0.3)	5 (0)	57 (0.3)
Junior trainee	*N* (%)	2715 (5.2)	990 (7.4)	363 (1.8)	1362 (7.2)
Senior trainee	*N* (%)	16,061 (30.8)	3273 (24.6)	5798 (29.3)	6990 (36.7)
Consultant	*N* (%)	33,287 (3.9)	9029 (67.9)	13,642 (68.9)	10,616 (55.8)

### Incidence of Nd:YAG capsulotomy

**Fig. 2:**
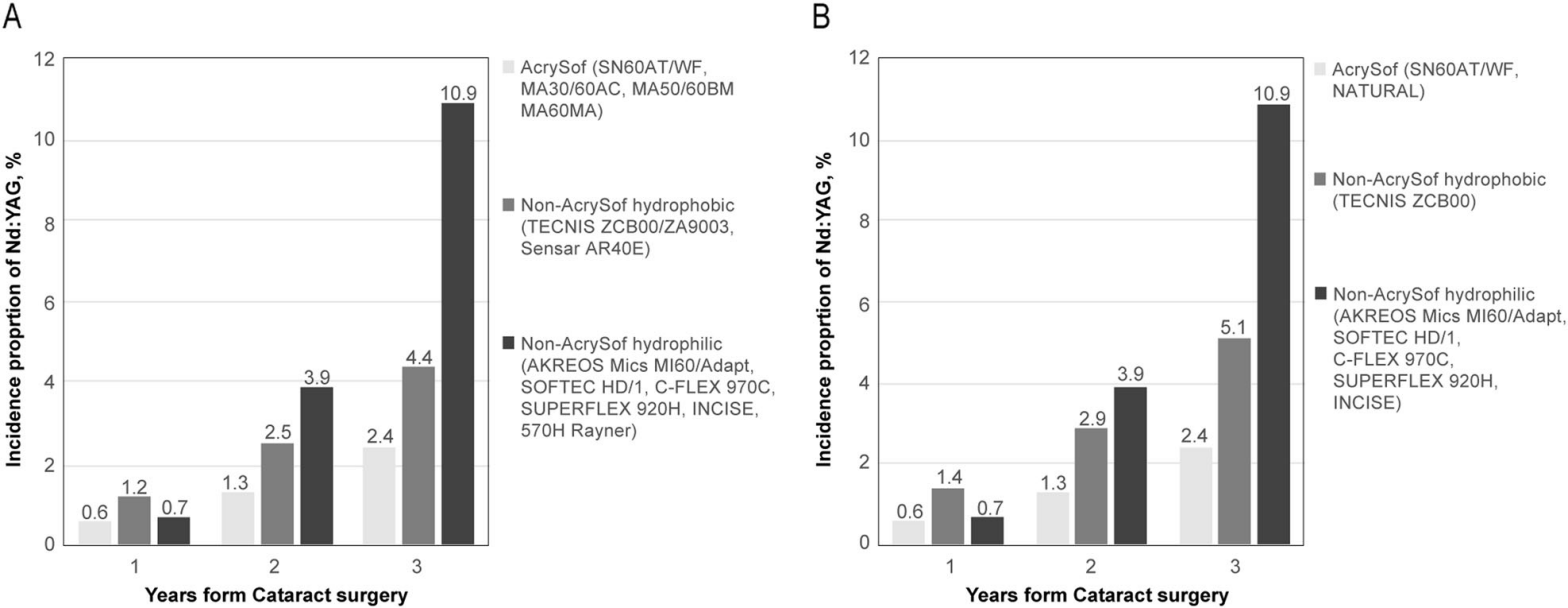
Incidence proportions of Nd:YAG capsulotomy at 1, 2 and 3 years following cataract surgery for eyes implanted with AcrySof, non-AcrySof hydrophobic and non-AcrySof hydrophilic IOLs. Figure 2a includes both single and multi-piece IOLs, Fig. 2b includes single-piece IOLs only

**Fig. 3:**
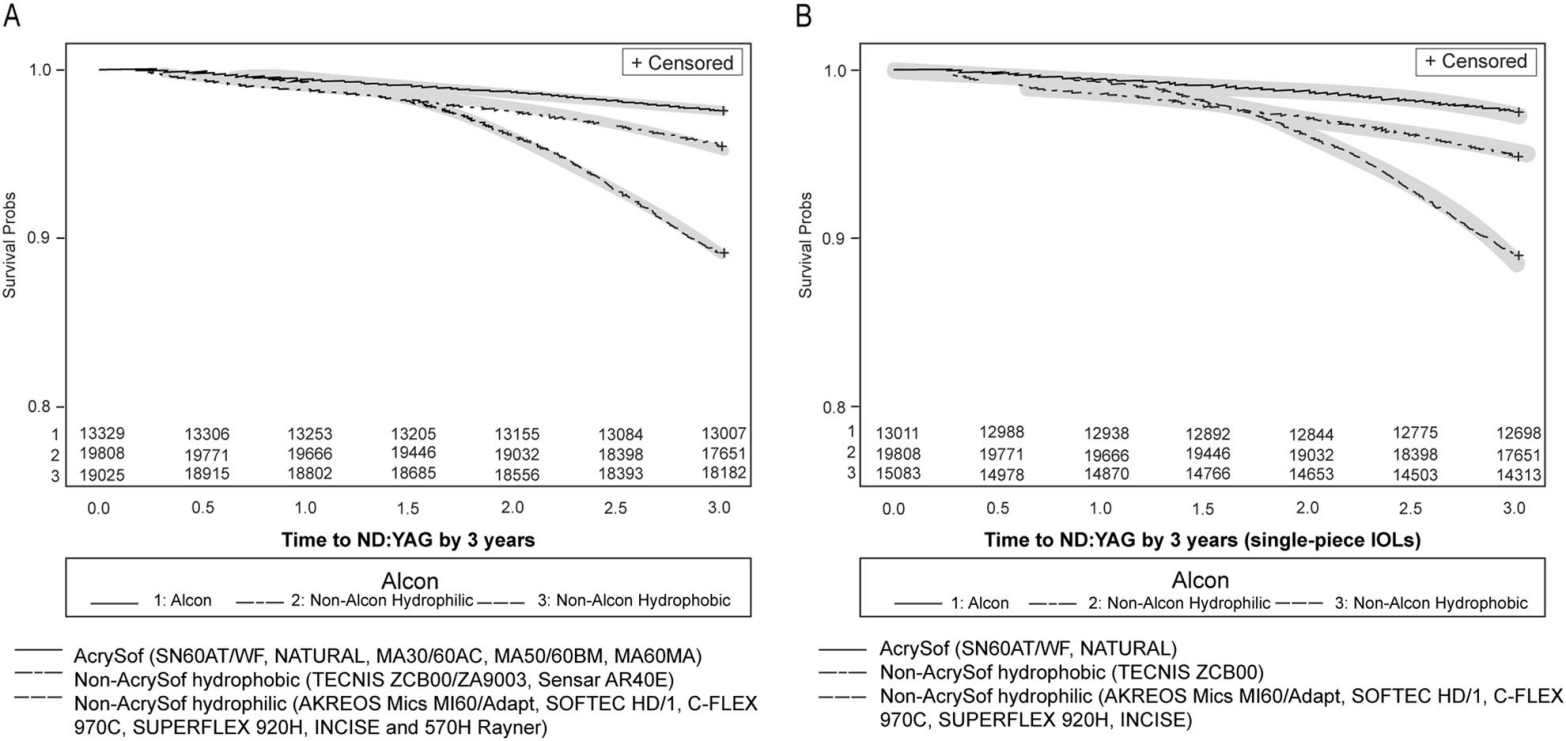
Kaplan–Meier survival plots of eyes implanted with AcrySof, non-AcrySof hydrophobic and non-AcrySof hydrophilic IOLs and not requiring Nd:YAG capsulotomy over 3 years following cataract surgery. Curves represent the survival estimates with number of subjects at risk and 95% Hall–Wellner Bands. Figure 3a includes both single and multi-piece IOLs, Fig. 3b includes single-piece IOLs only

At 3 years post-cataract surgery, the overall incidence proportion of Nd:YAG in the overall study cohort was 6.4%. The incidence proportions of Nd:YAG capsulotomy for each study cohort at 3, 2 and 1 years from cataract surgery are shown in Fig. 2, overall (Fig. 2a) and for the largest sub-cohort of single-piece IOLs (Fig. 2b). At 3 years, the Nd:YAG capsulotomy incidence was approximately two times lower for eyes implanted with AcrySof IOLs (2.4%, 2.2–2.7%, *N* = 322) compared to other hydrophobic acrylic IOLs (4.4%, 4.1–4.7%, *N* = 843) and four times lower compared to other hydrophilic acrylic IOLs (10.9%, 10.5–11.3%, *N* = 2 157). A similar trend was observed at 2 years (1.3%, 1.1–1.5% for Acrysof IOLs vs 2.5%, 2.2–2.7% for other hydrophobic acrylic IOLs and 3.9%, 3.6–4.2% for other hydrophilic acrylic IOLs (Fig. 2a)). At 1 year, the difference in incidence proportions was less evident for AcrySof (0.6%, 0.4–0.7%, *N* = 76) vs non-AcrySof hydrophilic IOLs (0.7%, 0.6–08%, *N* = 142), but the AcrySof cohort still recorded a 2 times lower incidence compared to the non-AcrySof hydrophobic cohort (1.2%, 1.0–1.3%, *N* = 223). These results were replicated in the single-piece IOLs sub-cohort (Fig. 2b). The Kaplan–Meier survival plots for eyes not undergoing Nd:YAG capsulotomy procedure over the 3 years follow-up are shown in Fig. 3.

### Odds ratios of undergoing Nd:YAG capsulotomy procedure

**Fig. 4:**
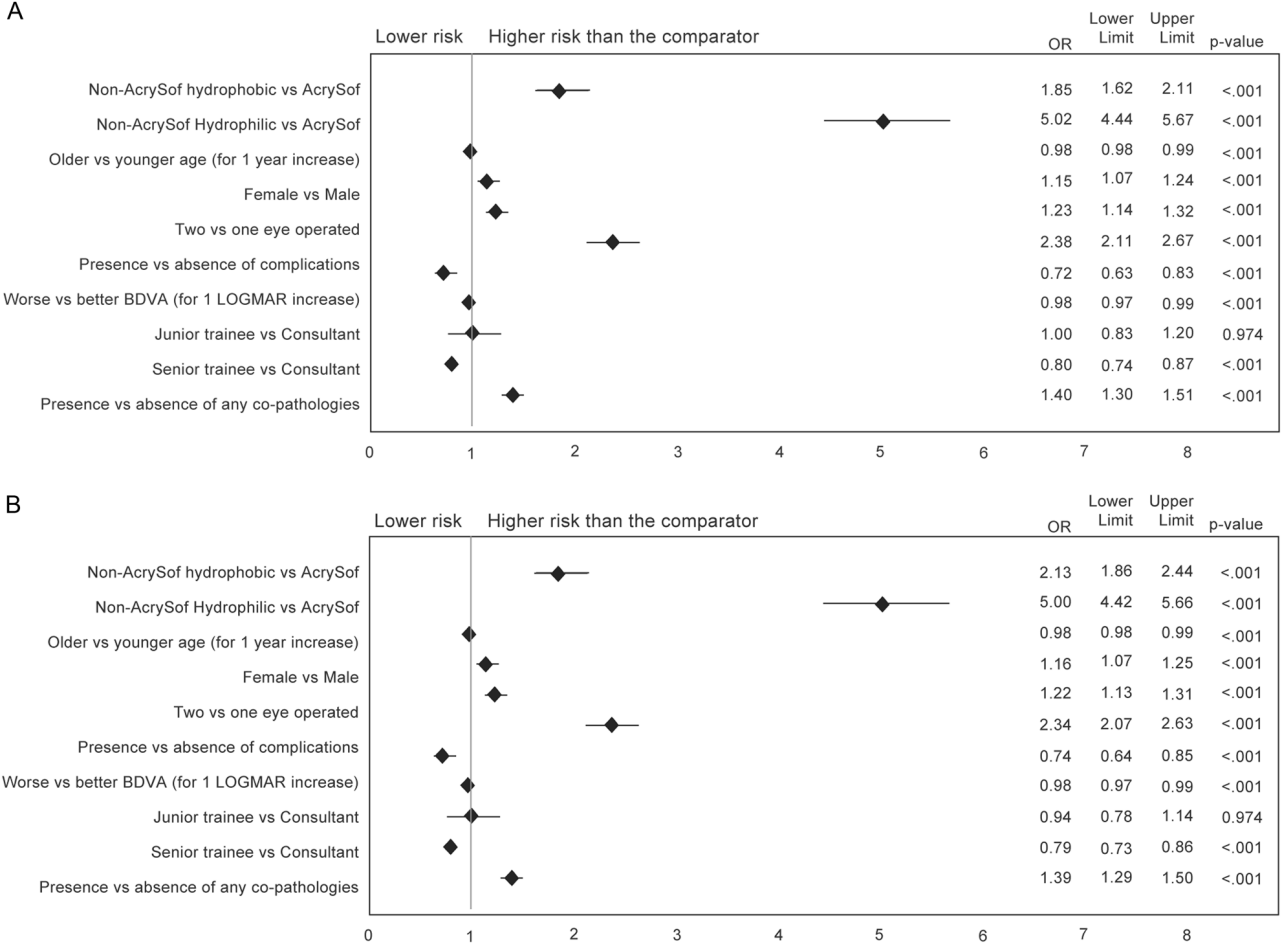
Adjusted ORs of undergoing Nd:YAG capsulotomy over 3 years from cataract surgery for eyes implanted with non-AcrySof hydrophobic and hydrophilic versus AcrySof IOLs. Multivariate logistic regression with level of significance = 0.05. ORs > 1 indicate that the comparator cohort is protective against Nd:YAG capsulotomy. **a** includes both single and multi-piece IOLs, **b** includes single-piece IOLs only

At 3 years post-cataract surgery, implantation with non-AcrySof hydrophilic acrylic IOLs was associated with an almost fivefold increased odds of undergoing Nd:YAG capsulotomy (unadjusted OR 4.94, 95% CI 4.38–5.56, *p* < 0.001) and implantation with non-AcrySof hydrophobic IOLs was associated with an approximate 2-fold increased odds (unadjusted OR 1.87, 95% CI 1.64–2.13, *p* < 0.001) compared to hydrophobic AcrySof IOLs. After adjusting for potential confounders, the OR did not substantially change and the odds of Nd:YAG capsulotomy at 3 years post-cataract surgery was still significantly higher (*p* < 0.001) for non-AcrySof hydrophobic and hydrophilic acrylic IOLs compared to AcrySof IOLs (Fig. 4a). The same trend was observed in single-piece IOLs only (Fig. 4b). The logistic regression showed that younger age, female gender, 2 versus 1 eyes operated, presence of any complications post-surgery, history of co-pathologies and lower IOL power are also independently associated with increased risk of Nd:YAG capsulotomy (Fig. 4). It was also observed that eyes with better Best Corrected Distance Visual Acuity (BCDVA) and those operated by consultant surgeons are at slightly higher risk than those operated by senior trainee (Fig. 4).

### Incidence of PCO

**Fig. 5:**
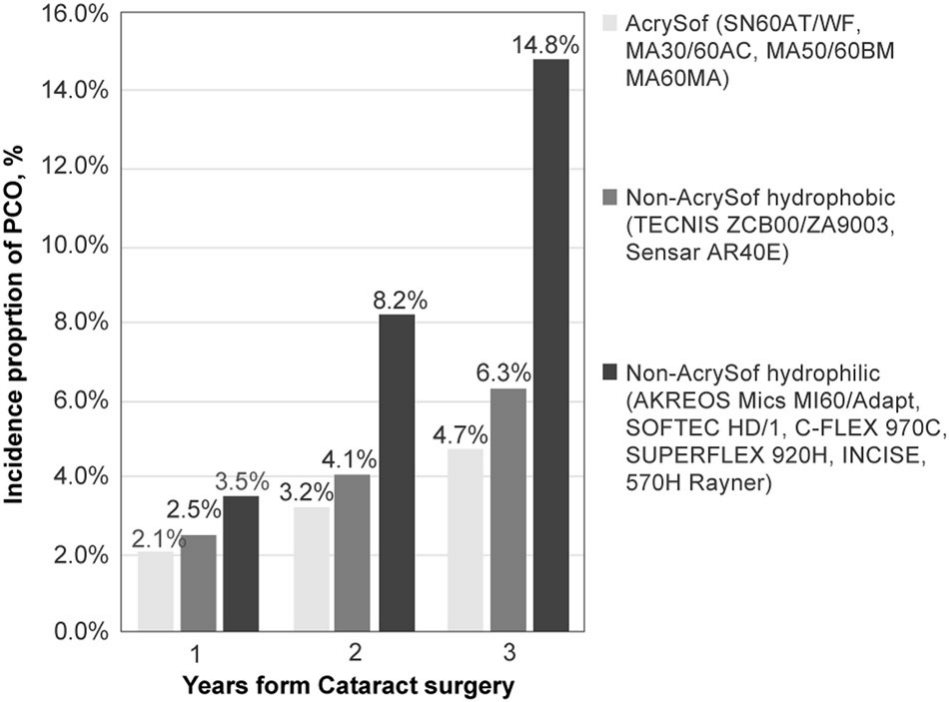
Incidence proportions of PCO at 1, 2 and 3 years following cataract surgery for eyes implanted with AcrySof, non AcrySof hydrophobic and non-AcrySof hydrophilic IOLs

At 3 years post-cataract surgery, the overall incidence proportion of PCO in the full cohort was 9.1%. At 1, 2 and 3 years respectively, incidence proportions (95% CI) of PCO were 2.1% (1.8–2.3%, *N* = 274), 3.2% (2.9–3.5%, *N* = 431) and 4.7% (4.3–5.0%, *N* = 625) for the AcrySof cohort; 2.5% (2.3–2.7%, *N* = 472), 4.1% (3.8–4.4%, *N* = 776) and 6.3% (6.0–6.7%, *N* = 1 205) for non-AcrySof hydrophobic cohort and 3.5% (3.2–3.7%, *N* = 690), 8.2% (7.8–8.6%, *N* = 1 621) and 14.8% (14.3–15.3%, *N* = 2 931) for non-AcrySof hydrophilic cohort (Fig. 5).

## Discussion

The current study found that approximately one tenth of cases develop PCO at three years post-cataract surgery and that more than half of them require a Nd:YAG capsulotomy procedure to treat PCO. This study demonstrated that IOL material and its surface properties can influence the incidence of Nd:YAG capsulotomy and PCO in acrylic IOLs. Our results confirmed previous evidence from smaller prospective studies [5–10] that incidence proportions of Nd:YAG capsulotomy and PCO are lower for hydrophobic compared to hydrophilic acrylic IOLs. This study further found that after 3 years from implantation, AcrySof IOLs are associated with significantly lower risk of requiring an Nd:YAG capsulotomy and have the lowest incidence of PCO compared to cohorts of other hydrophilic and hydrophobic acrylic IOLs. This protective effect was independent from potential confounders and was confirmed when the analysis was performed in the largest sub-group of single-piece IOLs.

Similar trends were found in a recent Swedish RWE study of 900 eyes that showed in comparison with a hydrophobic acrylic IOL with sharp posterior optic edge, a hydrophilic acrylic IOL was associated with twice the number of Nd:YAG capsulotomies over a 5-year period [25]. Another Swedish RWE study that followed 1527 patients for a mean duration of 3.5 years, also confirmed that the risk of Nd:YAG capsulotomy and PCO was significantly higher in hydrophilic IOLs compared with hydrophobic IOLs [26]. To our knowledge, this study is the largest analysis of the incidence of Nd:YAG capsulotomy and PCO performed to date.

It has been shown that different lens designs may also have an impact on the development of PCO following implantation with single-piece acrylic IOLs resulting in more Nd:YAG capsulotomies and PCO than multi-piece acrylic IOLs [23, 24]. To evaluate the effect of IOL materials without the confounding factor of haptic designs, we conducted the analyses in the sub-group of eyes implanted with single-piece IOLs (the most common IOL type used in our sample). The results of this sub-group analysis confirmed that single-piece Acrysof IOLs are associated with significantly lower risk of Nd:YAG capsulotomy and incidence of PCO vs. others.

Our study of longitudinal real-world data from over 50,000 procedures performed in seven different sites across the UK, with implantation of a variety of IOLs, provided a robust data set, offering real world clinical practice evidence beyond, randomised clinical trials which are conducted in a controlled environment. In order to reduce biases linked to non-randomisation, baseline characteristics were evaluated and statistical adjustment for potential confounders was performed. We also accounted for potential concerns of reliability for Nd:YAG capsulotomy rates in real-world retrospective studies compared to RCTs. In order to avoid the potential bias that patients did not return to the same clinic for PCO diagnosis and Nd:YAG treatment, we confirmed with all participating sites that each clinic would routinely conduct their own Nd:YAG capsulotomy procedures and had no other local NHS units routinely performing Nd:YAG capsulotomy on their patients. Another concern could be that Nd:YAG capsulotomy and PCO rates could be affected by practice differences between the clinics. The data did not contain information on the exact level of experience of each surgeon, capsulorrhexis size or location relative to the optic or type of initial cataract. This study represents an extremely large sample size of patients across multiple clinics and therefore reflect treatment practice in the real world, which is naturally variable unlike in clinical trials. Therefore, these factors were assumed to be evenly spread between the 3 groups.

We intended to control for site by including a site ID variable into the logistic regression model, however due to high correlation between lens type and site ID (sites generally use one or two lenses exclusively) site ID was not included in the final model to avoid collinearity. Removing site ID from the model did not substantially change the final results (data not shown).

Many studies on Nd:YAG capsulotomy and PCO rates have compared IOL material and designs, showing that a sharp square edge of the IOL is associated with lower PCO rates [1, 2]. It is believed that different IOLs have different properties at the square edges and that this could be related to the IOL material [2] which may explain the different rates of PCO observed in this study. However, the effect of other properties inherent in the AcrySof IOL or its material contributing to reduced incidence of PCO cannot be excluded. We were not able to analyse the effect of IOL configuration and edge morphology on the incidence of Nd:YAG capsulotomy and PCO within the different IOL cohorts. Despite this, we have demonstrated that AcrySof IOLs with a non-360° square (interrupted) edge and single-piece design had a lower incidence of PCO and significantly lower risk of undergoing an Nd:YAG capsulotomy procedure compared to single-piece acrylic hydrophilic and hydrophobic IOLs with a 360° square (continuous) edge. This finding contrasts with previous clinical paired-eye studies evaluating patients who had implantation of a non-AcrySof IOL with a continuous optic edge in 1 eye and an AcrySof IOL with an interrupted optic edge in the fellow eye [15, 27], where there was no statistical difference in the incidence of PCO between the two IOL types. Nixon et al. [27] reported a significantly lower incidence of PCO in eyes implanted with a continuous 360° square edge. The small sample size of these studies as well as other factors, such as lens models and the short follow-up duration, may explain discrepancies in findings.

Although the evaluation of potential other risk factors associated with PCO was not the focus of this study, adjusted multivariate analysis showed some characteristics are independently associated with increased odds of undergoing an Nd:YAG capsulotomy. These comprised presence of any complications post-surgery, history of co-pathologies (e.g. glaucoma, age-related macular degeneration, diabetic retinopathy) and, to a lesser extent, female gender and younger age, which is congruent with published evidence [2, 28, 29]. It is important to remember, however, that our study restricted the age to 65 years or older so our results are restricted to patients of this age cohort only. We also found that eyes implanted with lower-power IOLs were marginally, but significantly associated with increased risk of Nd:YAG capsulotomies, which may be related to increased myopia and axial length of the eye. However, a previous study has found that axial myopia did not significantly increase the area or incidence of PCO at 4 years, although the study had a much smaller sample size compared to this analysis [30]. Another possible explanation for this association could be related to a change in the shape of the square edge of the IOL. Finally, the authors could not find a clear explanation for the lower ORs recorded for eyes with higher BCDVA or for those operated by senior trainees compared to consultants. Future analyses should be targeted to investigate in more details the potential eye factors associated with the development of PCO independently from IOL properties.

The major strengths of this study are the longitudinal design and the large sample size, which allowed for strong statistical power in the comparative analyses and for the selection of a representative cohort of patients. The median age at cataract surgery and gender distribution in our study population were comparable to those reported in the National Ophthalmology Database (NOD) Audit 2016 Annual Report [31]. Other important strengths of the current analysis include the completeness of the data collection and consistency of recording across clinics which is mandated by the Medisoft EMR programme. This enabled us to collect a reliable set of parameters from multiple sites, allowing adjustment for potential confounders in the regression model. Finally, while retrospective studies can be limited by incomplete data collection and loss of patients for subsequent Nd:YAG capsulotomy, we minimised loss of data by only including those clinics where no alternative local pathways existed for patients to receive the Nd:YAG capsulotomy procedure following PCO.

Notwithstanding these strengths, the authors acknowledge some limitations to the study. Medisoft data only contains partial access to primary care data, thus limiting information on the patient’s entire medical history. Similarly, death may have been underreported. However, we believe that this would equally apply to all of the comparative cohorts and thus not greatly impact the findings. Finally, we selected a panel of clinics with high-quality Medisoft data entry for both cataract surgery and Nd:YAG capsulotomy, which may have biased the outcomes. To mitigate these biases, we selected NHS clinics having good data completeness in the Medisoft EMR, use of Medisoft EMR for the entire duration of the study (2010–2016), and from different geographical locations in the UK to account for regional variations in the clinical practices.

Based on the results of this study, in the 3 years following cataract surgery in NHS clinics in the UK, the risk of undergoing an Nd:YAG capsulotomy and the incidence of PCO are significantly lower when AcrySof IOLs are used, compared to other cohorts of hydrophobic and hydrophilic acrylic IOLs. Prevention of PCO is clinically important and beneficial to patients as this condition is associated with reduced visual performance, which is a significant morbidity in the elderly age group that this study analysed [32]. Poor vision is associated with depression, falls and reduced mobility in this age cohort [33, 34]. Furthermore, the reduction in the requirement of Nd:YAG capsulotomy to correct PCO can reduce healthcare costs and associated clinical risks [3, 35] The prevention of PCO and subsequent Nd:YAG capsulotomies could reduce the burden on the healthcare system concerning the healthcare resource use required for diagnosis, the procedure itself as well as any post-procedural monitoring, complications and any further treatments. Future studies should further evaluate this protective effect over longer follow-up durations.

## Disclaimer

The authors have no proprietary or commercial interest in any materials discussed in this article.

### Summary

#### What was known before

- Hydrophobic IOLs are associated with lower incidence of PCO and subsequent Nd:YAG capsulotomy procedure with respect to hydrophilic IOLs.

- IOL lens material has an important effect on the rates of PCO and Nd:YAG following implantation improvements in IOL material and design.

- Currently published work is mainly through clinical trials which are not feasible to provide estimates in large populations or over extended time horizons.

#### What this study adds

- This is the largest known study (over 52,000 eyes) validating the effect of IOL material on PCO and Nd:YAG rates.

- The study demonstrates that hydrophobic AcrySof IOLs are independently associated with a substantial lower odds of receiving an Nd:YAG procedure compared to other hydrophobic acrylic IOLs.

- The highly protective effect of AcrySof material remains valid when single-piece IOLs only are considered.
